# Individualisierung und Standardisierung in der Kopf-Hals-Pathologie

**DOI:** 10.1007/s00106-025-01627-y

**Published:** 2025-04-16

**Authors:** Andreas G. Loth, Peter J. Wild

**Affiliations:** 1 Universitätsklinikum Frankfurt, Klinik für Hals‑, Nasen- und Ohrenheilkunde, Goethe-Universität Frankfurt, Theodor-Stern-Kai 7, 60450 Frankfurt am Main, Deutschland; 2 Universitätsklinikum Frankfurt, Dr. Senckenbergisches Institut für Pathologie und Humangenetik, Goethe-Universität Frankfurt, Frankfurt am Main, Deutschland

**Keywords:** Medizinische Informatik, Biomarker, Präzisionsmedizin, Digitale Gesundheit, Computergestützte Biology, Medical informatics, Biomarkers, Precision medicine, Digital health, Computational biology

## Abstract

Individualisierung und Standardisierung sind scheinbar widersprüchliche Anforderungen in der Medizin. In der Tumorbehandlung von Kopf-Hals-Karzinomen haben beide Begriffe direkten Einfluss auf die Diagnostik, welche üblicherweise in pathologischen Instituten durchgeführt wird. Im vorliegenden Referat wird das Spannungsfeld zwischen technischen Untersuchungen, regulatorischen Anforderungen, strukturellen Änderungen durch Digitalisierung und dem Einzug der personalisierten Medizin beleuchtet. Ziel ist zum einen, durch ein Verständnis der Herausforderungen den interdisziplinären Austausch zu fördern, zum anderen sollen dem HNO-Arzt ganz praktisch die gängigen pathologischen Untersuchungen nähergebracht werden. Am Beispiel der Pathologie lässt sich so schlussendlich zeigen, dass die Standardisierung von Verfahren einer Verbesserung der individuellen Behandlung dient. Zugleich lassen sich aber auch die Herausforderungen ablesen: Trotz umfassender Regularien sowie einer Laborumgebung mit digitaler Unterstützung ist die Standardisierung sehr zeit- und kostenaufwendig. Sollen ähnliche Standardisierungsansätze beispielsweise in einem operativen Umfeld wie der HNO-Chirurgie umgesetzt werden, darf der Aufwand aufgrund der „menschlichen Komponente“ als gleichwertig oder höher eingeschätzt werden.

## Abstract (en)

Individualization and standardization are seemingly contradictory requirements in medicine. In the treatment of head and neck cancer, both terms have a direct influence on diagnostic procedures, which are usually carried out in pathology institutes. The current article examines the conflicting requirements arising from various technical analyses, regulatory requirements, structural changes due to digitalization, and the advent of personalized medicine. On the one hand, the goal is to promote interdisciplinary exchange by understanding the challenges and, on the other, to provide the otorhinolaryngologist with a practical understanding of the common and current pathological diagnostic tests. Using pathology as an example, it can be shown that standardization of procedures ultimately serves to improve individualized treatment. At the same time, however, the following challenges are also apparent: despite comprehensive regulations and a laboratory environment with digital support, standardization is very time consuming and costly. If similar standardization approaches are to be implemented in an operative environment such as, e.g., ENT surgery, the effort involved can be expected to be equivalent or higher due to the human factor.

## Geforderte Interdisziplinarität

Das Wort „Pathologie“ kommt wörtlich übersetzt aus den altgriechischen Wörtern „Pathos“, was Leiden oder Krankheit bedeutet und „Logos“, was mit Lehre übersetzt wird [1]. Die Pathologie bezeichnet also im ursprünglichen Sinne die Lehre von den Krankheiten. Daraus hat sich der klinische Bereich der Pathologie entwickelt, welcher sich v. a. mit der Beurteilung von Geweben im zellulären und subzellulären Bereich beschäftigt, um daraus Diagnosen von zugrunde liegenden Erkrankungen abzuleiten. Im klinischen Alltag der Hals‑, Nasen‑, Ohrenheilkunde liegen die häufigsten Berührungspunkte bei der Diagnose und Behandlung von Tumorerkrankungen im Kopf-Hals-Bereich. Weltweit kommt es jährlich zu geschätzten 660.000 Neuerkrankungen von Tumoren im Kopf-Hals-Bereich [2, 3].

**Abb. 1:**
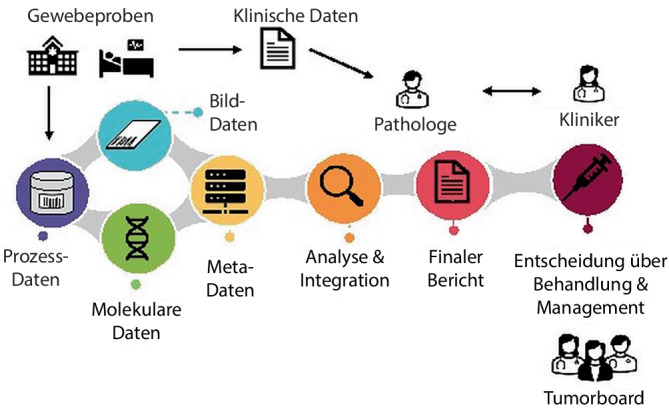
Ablauf der modernen Gewebediagnostik in der Pathologie mit Integration von molekularen und digitalen Daten im Rahmen der Präzisionsmedizin

Das Ziel aller Behandelnden bei solchen Erkrankungen ist, die möglichst passende Therapie für den jeweiligen Patienten zu finden. Hierbei spielt die Pathologie durch die Klassifizierung der Tumoren schon seit jeher eine große Rolle. Mit der Einführung jeder neuen Therapieform werden auch neue Biomarker gesucht, um die Gruppe der Patienten, auf welche diese Therapie angewendet werden kann, besser zu klassifizieren. Bei der Suche und Anwendung dieser Biomarker ergibt sich ein Spannungsfeld aus den technischen Anforderungen der Untersuchung einerseits und den regulatorischen Anforderungen des Gesetzgebers anderseits. Zudem spielen Faktoren wie eine großflächige strukturelle Änderung von lange eingeübten Arbeitsabläufen durch die Einführung der digitalen Pathologie sowie der interdisziplinäre Behandlungsansatz, beschrieben im Leitbild der Präzisionsmedizin, eine Schlüsselrolle im Alltag der Pathologen [4, 5]. Die Abb. 1 zeigt den Workflow der Gewebediagnostik und den Einfluss von Technologie und Datenverarbeitung in der modernen Präzisionsmedizin.

Um die geforderte Interdisziplinarität wirklich ausleben zu können, ist ein Grundverständnis des jeweils anderen Fachs unbedingt notwendig. Dieses Referat hat daher 2 Ziele: Das Erste besteht darin, HNO-Ärzten, auch nicht onkologisch tätigen, die Arbeitsweisen und aktuellen Spannungsfelder des Fachs Pathologie aufzuzeigen, um das gegenseitige Grundverständnis im interdisziplinären Austausch zu fördern. Das zweite Ziel ist es, einen für den HNO-Arzt verständlichen Überblick über häufig angewandte diagnostische Verfahren der Kopf-Hals-Pathologie sowie einen Einblick in aktuelle Neuentwicklungen zu geben.

**Abb. 2:**
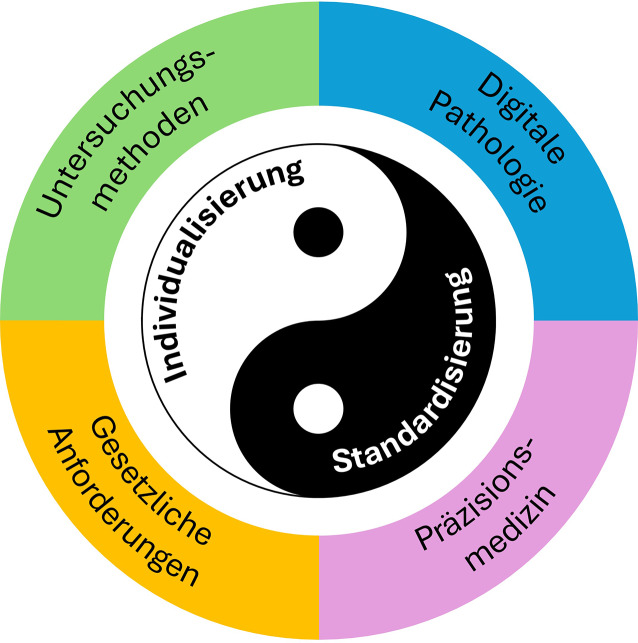
Einflussfaktoren auf Individualisierung und Standardisierung in der Pathologie

Um diese Ziele zu erreichen, wird auf die in Abb. 2 angegebenen Einflussfaktoren, nämlich diagnostische Verfahren, gesetzliche Regulation, Digitalisierung und Präzisionsmedizin jeweils gesondert eingegangen und die jeweilige Bedeutung für die Standardisierung und Individualisierung analysiert. In der Zusammenfassung werden die Ergebnisse der Abschnitte zusammengetragen und die Bedeutung für die HNO diskutiert.

## Diagnostische Verfahren in der Pathologie

Paul Ehrlich hat in der Rede zur Verleihung seines Nobelpreises in Medizin 1908 gesagt: „Die hier erzielten Ergebnisse basieren hauptsächlich auf histologischen Untersuchungen […]. Dennoch neige ich zu der Annahme, dass die Grenzen dessen, was das Mikroskop für uns tun kann und getan hat, nun näher rücken […] Jetzt […] müssen wir hierfür andere Untersuchungsmethoden finden.“ [6]. Er sollte recht behalten. Mittlerweile sind die diagnostischen Verfahren in der Pathologie so umfangreich, dass auch eine kurze Darstellung jedes einzelnen Verfahrens den Umfang dieser Arbeit weit übersteigen würde. Um die Frage der Standardisierung und Individualisierung in der modernen Medizin zu erörtern, sollen daher in diesem Kapitel 3 für die Kopf-Hals-Onkologie wichtige Bereiche und Verfahren weiter in die Tiefe gehend vorgestellt werden. Zunächst aus dem Bereich der Histopathologie, die Hämatoxylin-Eosin(HE-)Färbung, welche schon seit fast 150 Jahren das unangefochtene Arbeitspferd in der Pathologie ist. Zum zweiten aus dem Bereich der Immunfärbungen die Immunhistochemie, welche in der Diagnostik von Plattenepithelkarzinomen im Kopf-Hals-Bereich vor dem Hintergrund der Assoziation mit humanen Papillomaviren (HPV) und der verfügbaren Anti-PD-L1-Antikörper eine sehr wichtige Rolle spielt. Der dritte Bereich ist die DNA-Amplifikation und Sequenzierung. Hier ist v. a. das „next-generation sequencing“ (NGS) von höchster aktueller Bedeutung, weil dieses Verfahren in anderen onkologischen Disziplinen auch vor dem Hintergrund der aufkommenden „liquid biopsy“ schon jetzt eine herausragende Rolle spielen und vermutlich auch für die HNO immer wichtiger werden wird.

### Histopathologie und Gewebediagnostik

Das klassische Anfertigen und Färben von dünnen Gewebsschnitten auf Objektträgern und die Untersuchung mit den Lichtmikroskop bezeichnet man als Histopathologie. Es gibt zahllose Färbemethoden, aber die ohne Zweifel am häufigsten durchgeführte ist die HE-Färbung.

Die HE-Färbung ermöglicht eine schnelle und effiziente Beurteilung der Zell- und Gewebearchitektur und liefert eine übersichtliche Darstellung von Zellkernen, Zytoplasma und extrazellulären Komponenten. Dies ist essenziell für die Diagnostik von Tumoren, entzündlichen Erkrankungen und degenerativen Veränderungen.

#### Geschichte.

Die HE-Färbung ist eine der ältesten Färbungen in der Histopathologie. Der Farbstoff Hämatoxylin wurde ursprünglich aus dem Holz des „Blut- oder Blauholzbaums (lat. *Haematoxylum campechianum*)“ gewonnen [7]. Dieser Baum wächst in Mittelamerika und wurde 1502 durch spanische Seefahrer erstbeschrieben. Die indigenen Völker Mittelamerikas nutzten den Farbstoff in der Textilfärbung und im medizinischen Bereich. In den ersten Jahrhunderten nach der Entdeckung lag das primäre Interesse ebenfalls in der Gewinnung für die Textilindustrie, aus dem ein großer Handel mit dem entsprechenden Holz resultierte [7].

Erste Erfahrungen im Bereich der histopathologischen Färbung werden Wilhelm von Waldeyer-Hartz und Franz Böhmer in den 1870er-Jahren zugeschrieben, wobei diese noch ohne die klassische Gegenfärbung mit Eosin erfolgten [7]. In der gleichen Dekade wurde der Farbstoff Eosin entwickelt. Die Färbung, welche saure zytoplasmatische Inhalte färbt, war eine technische Entwicklung von Heinrich Caro, einem Chemiker, der zu diesem Zeitpunkt die Forschungsabteilung von BASF in Ludwigshafen leitete [8]. Die Kombination beider Färbungen wurde in den 1880er-Jahren zum ersten Mal von Moisej Alexandrowitsch Wissowzky beschrieben und setzte sich schnell als eine der häufigsten Standardfärbungen durch [9].

#### Methode.

Der grundlegende Ablauf der HE-Färbung besteht aus den folgenden hintereinander geschalteten Prozessschritten [10, 11]:

- Entwachsen
- Hydrierung
- Hämatoxylin-Färbung
- Differenzierung
- Bläuen
- Eosin-Färbung
- Dehydrierung
- Klärung
- Eindecken

*Entwachsen*: Wenn ein Paraffinschnitt gefärbt werden soll, ist das Gewebe mit Paraffin durchtränkt. Um dieses färbbar zu machen, muss das Paraffin bei diesem Zwischenschritt entfernt werden. Hierzu wird i. d. R. Xylol verwendet.

*Hydrierung*: Das Xylol muss ausgewaschen und das Gewebe für wässrige Farbstoffe durchdringbar gemacht werden. Hierzu wird eine absteigende Alkoholreihe mit anschließender Spülung mit Wasser verwendet.

Bei der *Hämatoxylin-Färbung* geht es um das eigentliche Durchdringen des Gewebes mit dem Farbstoff. Zur histologischen Färbung wird zudem ein sog. Beizmittel benötigt, welches eine Brücke zwischen Gewebe und Farbstoff herstellt. Hier werden i. d. R. Metallsalze verwendet.

Bei der *Differenzierung* wird der überschüssige Farbstoff mit einer Säure ausgewaschen. Hier können verschiedene Säuren zu unterschiedlich starken Hintergrundfärbungen führen, da jeweils unterschiedliche Mengen an Farbstoff ausgewaschen werden.

*Bläuen*: Hämatoxylin selbst ist nicht farbgebend, es muss zur Färbung zu Hämatin oxidiert werden. Erfolgt die Oxydation durch Luftsauerstoff, dauert der Prozess etwa 6–8 Wochen. Ein Beifügen von Oxidationsmittel wie Natriumiodat, Wasserstoffperoxid, Quecksilberoxid oder Kaliumpermanganat verringert die Reaktionsdauer auf Sekunden bis Minuten.

*Eosin* färbt fast alles, was durch Hämatoxylin nicht gefärbt wird, und ist daher die ideale Gegenfärbung.

*Dehydrierung*: Überschüssiges Wasser muss wieder aus dem Gewebe entfernt werden. Dies geschieht mit 100 % Alkohol.

*Klärung* bedeutet Entfernung des Alkohols, damit der Klebstoff für die Deckgläschen hält.

*Eindecken* bezieht sich auf das Festkleben des Deckgläschens.

Auch wenn die Grundschritte i. d. R. gleich bleiben, so kann doch jeder einzelne dieser Parameter verändert werden. Allein durch die Dauer der einzelnen Schritte, die Anzahl der Wiederholungen, die zur Differenzierung verwendete Säure oder das Beizmittel werden sehr große Unterschiede in der Farbgebung und Kontrastierung erreicht [10]. Entsprechend existieren unzählige Färbeprotokolle, in denen die einzelnen Schritte und verwendeten Materialien dargestellt werden. Diese Unterschiede haben auch einen großen Einfluss auf die Standardisierung, welcher zu einem späteren Zeitpunkt ausführlicher diskutiert werden soll [10].

#### Bedeutung in der Kopf-Hals-Pathologie.

Aufgrund der langen Erfahrung und der genannten Vorteile wird die HE-Färbung bei fast allen Fragestellungen in der Kopf-Hals-Pathologie als Erstes angefertigt und beurteilt. Zusammen mit den klinischen Angaben kann so in vielen Fällen bereits eine Diagnose gestellt werden, welche dann mit anderen Verfahren, wie z. B. der Immunhistochemie, validiert wird. Konkret wird die HE-Färbung an Zytologien, Biopsien, Schnellschnitten und Resektionspräparaten eingesetzt. Aktuell sind Kliniker mit der Beurteilung solcher Schnitte gewöhnlich nicht vertraut, da zur Betrachtung ein Lichtmikroskop notwendig ist und die einzelnen Schnitte i. d. R. nicht physisch zu Verfügung stehen. Dies wird sich mit einer flächendeckenden Einführung der digitalen Pathologie ändern. Zukünftig könnten dann in Tumorkonferenzen analog zu den radiologischen Bildern die histologischen Bilder von den beurteilenden Pathologen demonstriert und interdisziplinär diskutiert werden. Denn obgleich der Pathologe auch in Zukunft der unbestrittene Experte auf dem Feld der histologischen Diagnostik sein wird, so ist doch die Aussage des Operateurs beispielsweise bei der Beurteilung von Randschnitten in vielen Fällen hilfreich.

#### Individualisierung vs. Standardisierung.

Die Standardisierung hat im Bereich der HE-Färbung schon mit ihrer Entwicklung Einzug gehalten. Wie bereits beschrieben, machen schon kleine Änderungen in der Dauer, den Wiederholungen oder den Zusammensetzungen der verwendeten Inhaltsstoffe große Unterschiede bei dem Ergebnis der Färbung [10, 11]. Um immer das gleiche Färbeergebnis zu haben, wurden Färbeprotokolle entwickelt, welche die Schritte genau dokumentieren und darstellen. In der Praxis zeigen sich aber bei der händischen Färbung Unterschiede zwischen den ausführenden Personen auch bei gleichem Protokoll. Etwas Abhilfe schaffen hier Färbeautomaten, welche die entsprechenden Schritte nach vorprogrammierten Zeitabläufen immer gleich durchlaufen. Noch größere Unterschiede gibt es selbstverständlich bei Verwendung von unterschiedlichen Protokollen. Hier gibt es keine bundes- oder weltweit einheitlichen Standards. Dies hat bei der Beurteilung durch einen menschlichen Pathologen nur bedingte Auswirkung, da das Gehirn diese Unterschiede extrapolieren kann. Von Bedeutung werden die Unterschiede allerdings, wenn man beispielsweise die Zellkerne mittels automatischer Segmentierung durch ein Bilderkennungsprogramm auswerten will [12]. Hier spielen die Kontraste und Farbgebung eine entscheidende Rolle. Da diese Art der Auswertung v. a. im Forschungsbereich immer wichtiger wird, wird vermutlich auch eine weitere Standardisierung zwischen Laboren zunehmen [13].

### Immunfärbungen

Die Immunfärbung bezeichnet ein breites Feld der Färbungen, bei dem Antikörper dafür genutzt werden, selektiv Proteine in der jeweiligen Untersuchungsmethode (Mikroskopie, Fluoreszenzmikroskopie usw.) sichtbar zu machen. Ursprünglich wurde der Terminus zur Bezeichnung der „Immunhistochemie“ genutzt. Mittlerweile umfasst er aber ein breites Spektrum an Methoden, die alle antikörperbasierte Färbemethoden nutzen. In der HNO wird die Immunhistochemie bei der Beurteilung sowohl von Speicheldrüsentumoren als auch Plattenepithelkarzinomen genutzt. Bei der Beurteilung von Plattenepithelkarzinomen ergeben sich abhängig von der Immunhistochemie konkrete therapeutische Konsequenzen. Daher wird im folgenden Abschnitt auf die Immunhistochemie und 2 Färbungen, welche bei der Beurteilung von Tumoren im HNO-Bereich (p16 und PD-L1) von Bedeutung sind, detailliert eingegangen. Da bei der Immunhistochemie die zentrale Komponente eine spezifische Antikörperbindung ist, sollen unter diesem Kapitel auch die „antibody-drug conjugates“ (ADC) in der Kopf-Hals-Pathologie abgehandelt werden, obwohl die Antikörper hier nicht mehr diagnostisch, sondern schon therapeutisch genutzt werden.

### Immunhistochemie

In der Immunhistochemie (IHC) wird das Prinzip der spezifischen Bindung zwischen Antikörpern und Antigen genutzt, um ausgewählte Antikörperstrukturen sichtbar zu machen. Es gibt hier die direkten und die indirekten Möglichkeiten. Bei der direkten Färbung wird ein Antikörper, welcher gleichzeitig einen Marker trägt, der von der jeweiligen Nachweismethode (beispielsweise Lichtmikroskopie oder Fluoreszenzmikroskopie) erkannt werden kann, auf das Gewebe gegeben. Bei der indirekten Methode wird zunächst ein Primärantikörper verwendet, welcher an das Epitop des nachzuweisenden Antigens bindet. In einem zweiten Schritt wird ein Marker hinzugegeben, der an den Primärantikörper bindet und in der jeweiligen Untersuchungsmethode nachgewiesen werden kann. Neben (fluoreszierenden) Farbsignalen dienen auch Ergebnisse einer Enzym-Substrat-Reaktion der Auswertung, wenn an den Sekundärantikörper ein Enzym gekoppelt ist.

#### Geschichte.

Als Vorläufer der heutigen Immunhistochemie gelten die Arbeiten zu „Immunfluoreszenz“, welche 1941 von Albert Hewett Coons, Ernest Berliner, Norman Jones and Hugh J Creech entwickelt wurde und im ersten praktischen Einsatz Pneumokokken-Antigene in Gewebe nachgewiesen hat [14, 15]. Ein entscheidender Fortschritt war die Einführung enzymatischer Marker wie Peroxidase in den 1960er-Jahren, wodurch die Methode sicherer und praktikabler wurde [16]. In den 1970er-Jahren revolutionierten monoklonale Antikörper, entwickelt von Köhler und Milstein, die IHC. Diese Antikörper bieten eine hohe Spezifität und Reproduzierbarkeit und eröffneten neue Möglichkeiten zur Identifikation zellulärer und subzellulärer Antigene [16, 17].

#### Methode.

Ähnlich wie bei der beschriebenen HE-Färbung gibt es zahllose verschiedene Möglichkeiten, eine immunhistochemische Färbung durchzuführen. Eine genaue Beschreibung all dieser Verfahren würde den Rahmen dieser Arbeit übersteigen. Entsprechend werden im folgenden Abschnitt die Grundlagen für eine typische indirekte immunhistochemische Färbung mit einem monoklonalen Antikörper beschrieben [18–20]:

- Probenvorbereitung
- Entwachsung
- Antigenwiederherstellung
- Hemmung/Blockade der probeneigenen Zielaktivität
- Blockierung von unspezifischen Bindungsstellen
- Immunfärbung
- Gegenfärbung

*Probenvorbereitung*: Es sollten möglichst dünne Schnitte (2–5 µm) verwendet werden. Diese können von fixierten oder kryokonservierten Gewebe stammen. Die dünnen Gewebsschnitte müssen idealerweise auf dem Objektträger haften, deswegen werden die Objektträger i. d. R. mit 3‑Aminopropyltriethoxysilan (APTS) oder Poly-L-Lysin behandelt. Beide Lösungen lassen Aminogruppen auf der Glasoberfläche zurück, an denen das geschnittene Gewebe besser binden kann.

*Entwachsung*: Wenn fixiertes und in Paraffin eingebettetes Gewebe verwendet wird, muss das Paraffin entfernt werden, dies wird, analog zur HE-Färbung, oft mit Xylol durchgeführt.

*Antigenwiederherstellung*: Ein weiteres Problem für die IHC ist, dass bei der Formaldehydfixierung die Proteine in Gewebeproben durch Methylenbrücken kovalent vernetzen werden. Dies kann in manchen Fällen verhindern, dass die Primärantigene an den geplanten Epitopen binden. In so einem Fall muss nach dem Entwachsen das „epitope retrieval“ oder „antigen retrieval“ durchgeführt werden, damit der Primärantikörper wieder binden kann. Dies kann z. B. durch Erhitzen in verschiedenen Pufferlösungen, mit verschiedenen pH-Werten oder durch ein proteolytisches Enzym wie Pepsin, Trypsin oder Proteinase K geschehen.

*Hemmung/Blockade der probeneigenen Zielaktivität*: Wenn die gewählten Antikörper oder Nachweismethoden auf Verfahren beruhen, die auch natürlich in der Probe vorkommen können, wie beispielsweise die Nutzung von Biotin oder der alkalischen Phosphatase, dann sollten diese probeneigenen Proteine/Enzyme vor der IHC gehemmt oder blockiert werden, um falsch-positive Ergebnisse oder hohe Hintergrundfärbungen zu vermeiden.

*Blockierung von unspezifischen Bindungsstellen*: Um die Ergebnisse zu verbessern, können abhängig vom verwendeten Antikörper, mögliche unspezifische Bindungsstellen vor dem Färben blockiert werden.

Für die *Immunfärbung* kommen die beschriebenen direkten oder indirekten Methoden zum Einsatz. Die Antikörper werden i. d. R. in Pufferlösungen auf die Schnitte aufgetragen, um eine gute Verteilung zu gewährleisten. Wie bei der herkömmlichen Histologie ist auch hier ein ausgiebiges Spülen der Proben notwendig, um die nicht gebundenen Antikörper zu entfernen und somit Ergebnisverfälschungen vorzubeugen.

Die Verwendung von *Gegenfärbungen* soll einen Kontrast zur Primärfärbung bieten. Diese sind i. d. R. zellstrukturspezifische und meist einstufige Färbungen. Beispiele sind Hämatoxylin, Eosin, Nuclear Fast Red und Andere.

#### Bedeutung in der Kopf-Hals-Pathologie.

Die Immunhistochemie ist aus der Diagnostik der Kopf-Hals-Karzinome nicht mehr wegzudenken. In den meisten Fällen wird sie allerdings momentan noch bei der Diagnosestellung genutzt. In 2 Fällen hat sie jedoch direkten Einfluss auf die Therapiemöglichkeiten von Plattenepithelkarzinomen im Kopf-Hals-Bereich. Das ist zum einen beim Screening auf den HPV-Status und zum anderen beim Feststellen der PD-L1-Expression. Aus diesem Grund wird auf diese Bereiche im folgenden Abschnitt vertiefend eingegangen.

#### p16-Immunhistochemie im Kontext von HPV-assoziierten Tumoren.

Bei der Beurteilung von Kopf-Hals-Tumoren ist die Immunhistochemie nicht mehr wegzudenken. Die p16^INK4a^-(abgekürzt p16)-Immunhistochemie hat mittlerweile einen hohen Einfluss bei der Diagnose von HPV-positiven Tumoren. Um die 2000er-Jahre mehrten sich die Publikationen, die einen Zusammenhang zwischen HPV und Tumoren im Kopf-Hals-Bereich, v. a. im Oropharynx, feststellten und plausible Pathomechanismen darlegten [21–27]. Die Inzidenz variiert sehr stark zwischen verschiedenen Regionen. Je nach Studie werden etwa 40–60 % der Oropharynxkarzinome mit HPV assoziiert [28–30]. Dabei wird die Mehrheit dieser Tumoren durch HPV16 verursacht [31]. Diese Bedeutung wird auch in den Leitlinien aufgegriffen. Hier sind HPV-positive Oropharynxkarzinome seit der 8. Ausgabe der TNM-Klassifikation im Jahr 2017, welche vom American Joint Committee on Cancer (AJCC) und der Union for International Cancer Control (UICC) herausgegeben wird, eine eigene Entität [32]. Aktuell werden gerade auf den HPV-Status angepasste Therapieregime entwickelt und validiert, sind aber noch nicht einheitlich in der Leitlinie verankert [33–35]. Gegenwärtig ist der Goldstandard in der HPV-Diagnostik als direkte Methode der Nachweis von HPV-DNA oder bei den indirekten Methoden die p16-Immunhistochemie. Auf diesen Goldstandard im indirekten Nachweis soll im Folgenden näher eingegangen werden.

Das exprimierte p16-Protein ist in der Zelle die physiologische Stressantwort und verhindert über die Komplexbildung mit Cyclin D und den cyclinabhängigen Kinasen 4 oder 6 (CDK4 oder CDK6) die Phosphorylierung des Retinoblastom-Proteins und damit letztlich den Eintritt in die Synthesephase (S-Phase) der Zellteilung [36]. Bei der HPV-induzierten Onkogenese weist p16 ebenfalls eine Überexpression auf und kann somit als indirekter Biomarker für eine HPV-Infektion dienen. Dies führt dazu, dass der Immunhistochemie auf p16 im Kopf-Hals-Bereich eine besondere Bedeutung, v. a. bei Plattenepithelkarzinomen des Oropharynx, zukommt [37]. In einer Metanalyse mit 7654 Patienten für die p16- und HPV-Daten vorlagen zeigte sich, dass bei 3805 Patienten p16 positiv war, davon ließ sich bei 415 (9,1 %) Patienten keine HPV-Infektion nachweisen, sie galten somit als falsch-positiv. Der Anteil der falsch-negativen Ergebnisse (p16-negativ, HPV+) lag hingegen bei 289 (3,7 %). Aufgrund der hohen Sensitivität, der vergleichsweise niedrigen Kosten und der schnellen Durchführbarkeit hat sich die p16-Immunhistochemie also in der Kopf-Hals-Pathologie als Screeningverfahren für eine Assoziation der Tumoren mit HPV etabliert und wird vom College of American Pathologists routinemäßig bei Proben von Plattenepithelkarzinomen des Kopf-Hals-Bereichs empfohlen [38]. In Zukunft könnte dieses Verfahren aber Konkurrenz durch direkte Nachweismethoden bekommen. Weitere Entwicklungen sind v. a. im Bereich der „Liquid-Biopsie“, bei denen der Nachweis im Blut erfolgen soll, zu sehen [39].

#### Programmed-Death-Ligand-1-Diagnostik.

„Programmed death ligand-1“ (PD-L1) ist zusammen mit dem verwandten „programmed death ligand-2“ (PD-L1) der Ligand des „programmed death receptor 1“ (PD-1). PD‑1 wird auf Immunzellen, v. a. auf T‑Zellen, B‑Zellen und myeloischen Zellen, exprimiert [40]. Der PD-L1/PD-1-Komplex hat verschiedene physiologische Aufgaben. Zum einen ist er zwingend für die Entwicklung einer Immuntoleranz notwendig. Mit diesem Signalweg wird die negative Selektion von autoreaktiven Lymphozyten in den primären und sekundären lymphatischen Organen sichergestellt [41]. Außerdem wird durch den Signalweg die „T-cell exhaustion“ bei chronischen Entzündungen vermittelt. Kommt es bei solchen chronischen Entzündungen zu einer kontinuierlichen Antigenexposition der T‑Zellen, sorgt PD-L1/PD‑1 dafür, dass die T‑Zellen nicht dauerhaft hochaktiv sind und so zu Gewebszerstörung führen [42]. Bei der Tumorpathogenese findet sich eine anhaltende Hochregulierung von PD‑1 häufig in tumorinfiltrierenden Lymphozyten, wo die PD-L1-Expression von Tumorzellen ausgenutzt wird, um eine Zerstörung durch das Immunsystem zu vermeiden [40, 43, 44].

Die Expression von PD-L1 entweder im Tumor oder in infiltrierenden Immunzellen wurde bei einer Vielzahl von Tumoren überwiegend durch Immunhistochemie nachgewiesen [40]. Sie hat sowohl prognostische als auch therapeutische Bedeutung. Für Plattenepithelkarzinome im Kopf-Hals-Bereich wurde beobachtet, dass eine Überexpression von PD-L1 eine schlechtere Prognose und eine erhöhte Chemoresistenz aufweist [45, 46].

Therapeutische Bedeutung hat die Expression von PD-L1, weil es für verschiedene Tumorentitäten zugelassene Checkpointinhibitoren gibt, welche die Therapie mit monoklonalen Antikörpern maßgeblich beeinflussen können. In der Therapie von Plattenepithelkarzinomen des Kopf-Hals-Bereichs sind hier die monoklonalen Antikörper Nivolumab und Pembrolizumab zu nennen, welche vom gemeinsamen Bundesausschuss 2017 und 2019 für Rezidive bzw. Metastasen nach oder während platinhaltiger Chemotherapie zugelassen wurden [47, 48]. Diese beiden Antikörper sind neben dem im EXTREME-Schema angewendeten Anti-EGFR die einzigen aktuell in Deutschland zugelassenen Antikörper in der Therapie von Plattenepithelkarzinomen des Kopf-Hals-Bereichs. Auch wenn hier eine deutliche Zunahme der Immuncheckpointinhibitoren erwarten werden kann [49], verdeutlicht dies die Bedeutung der PD-L1-Testung für die Therapie der Patienten.

### „Antibody-drug conjugates“

„Antibody-drug conjugates“ (ADC) sind eine vielversprechende Therapieoption in der Tumortherapie. Hierbei werden monoklonale Antikörper über einen spezifisch entwickelten „chemical linker“ an zytotoxische Medikamente gekoppelt und so die hohe Spezifität von Antikörpern mit der Wirksamkeit von Chemotherapeutika kombiniert. So können zielgerichtet Tumorzellen angegriffen und dabei gesundes Gewebe geschont werden [50]. Aktuell sind 13 ADC in der Behandlung von hämatologischen sowie soliden Tumorerkrankungen zugelassen, und über 100 befinden sich in der Erprobungsphase [51] In Studien zeigen ADC bei HNO-Tumoren Potenzial, insbesondere bei schwer behandelbaren Subtypen wie Plattenepithelkarzinomen. Die Entwicklung von ADC erfordert eine präzise Auswahl von Zielantigenen, die spezifisch auf HNO-Tumorzellen exprimiert werden [52]. Herausforderungen bei ADC umfassen die Resistenzentwicklung und potenzielle Nebenwirkungen, was kontinuierliche Forschung erfordert [53].

#### Individualisierung vs. Standardisierung.

Der IHC-basierte Nachweis der PD-L1-Expression wird jedoch durch präanalytische und analytische Variabilität eingeschränkt. Hierzu gehören u. a. die Heterogenität bei Antikörperklonen, Bewertungsmethodik und intrinsischer biologischer Variation bei der PD-L1-Expression aufgrund der Art der analysierten Probe (chirurgische Resektion vs. Biopsie, Primärtumor vs. Metastase, fixiert vs. kryokonserviert) sowie des vorherigen Behandlungsstatus [40, 54, 55]. Da es bei diesen Untersuchungen um die definitive Entscheidung geht, ob eine zielgerichtete Therapie erfolgt oder nicht, sind für sämtliche Biomarker reproduzierbare Scoring-Systeme entwickelt worden, die eine einheitliche Auswertung und zwischen unterschiedlichen Laboren vergleichbare Ergebnisse gewährleisten [56]. Aktuell werden in Deutschland bei dieser Fragestellung 2 Scoring-Systeme verwendet. Zum einen der Combined Positive Score (CPS) und zum anderen der Tumor Positive Score (TPS) [33]. Den CPS errechnet man, indem man das Verhältnis der PD-L1-positiven Tumor- *und* Immunzellen (Lymphozyten und Makrophagen) zu allen Tumorzellen berechnet und mit 100 multipliziert [33]. Der TPS ist der prozentuale Anteil der Tumorzellen, welche PD-L1-positiv sind [33]. Gerade wenn neue Verfahren in der Therapie entwickelt werden, ist eine verlässliche Vergleichbarkeit der diagnostischen Methoden wichtig. Da eine technische Umsetzung von standardisierten Ergebnissen sehr schwierig ist, ist der Einsatz von interdisziplinär entwickelten, verhältnisbasierten Scoring-Systemen wichtig und wird vermutlich in der Zukunft noch wichtiger werden.

### DNA-Amplifikation und Sequenzierung

Die Gesamtheit der genetischen Information eines Organismus bezeichnet man als Genom. Das menschliche Genom besteht aus etwa 3 Mrd. Basenpaaren und ist in 23 Chromosomenpaaren organisiert, wobei es 24 Chromosomen gibt [57, 58]. Die rund 20.000 bis 25.000 Gene können in verschiedene Kategorien unterteilt werden, darunter strukturelle Gene, die für die Herstellung von Zellstrukturen verantwortlich sind, und regulatorische Gene, die die Aktivität anderer Gene steuern.

Zur Analyse dieser großen Mengen an genetischer Information, nicht nur im menschlichen Genom, sind im Wesentlichen 3 Verfahren notwendig. Die erste notwendige Entwicklung war, die DNA zu Analysezwecken zu vervielfältigen, was durch die Einführung der „polymerase chain reaction“ (PCR) gelang. Mit der PCR kann man beliebige Stücke einer DNA in Zyklen kopieren. Es kommt bei jedem Zyklus der PCR zu einer Verdopplung der ausgewählten und im Reaktor vorhandenen DNA-Stücke, sodass die Ziel-DNA exponentiell vermehrt wird. Den Prozess, die vorhandene DNA bei dem Vorgang der Replikation zu analysieren, also die Abfolge der Basenpaare zu erfassen, nennt man Sequenzierung. Die klassische DNA-Sequenzierung umfasst hauptsächlich 2 Verfahren: die Sanger-Sequenzierung und die Maxam-Gilbert-Sequenzierung. Beide Methoden waren entscheidend für die frühe Entschlüsselung des menschlichen Genoms, wobei die Sanger-Sequenzierung aufgrund ihrer Einfachheit und Genauigkeit breitere Anwendung fand.

### „Next-generation sequencing“

Die Weiterentwicklungen der klassischen, bereits beschriebenen Sequenzierungen werden als „next-generation sequencing“ (NGS) bezeichnet. Es existieren verschiedene Möglichkeiten und unterschiedliche Techniken, denen allen gemeinsam ist, dass sie Millionen kurzer DNA-Fragmente gleichzeitig sequenzieren. Diese kurzen Fragmente werden anschließend mithilfe computerbasierter Analysen anhand eines Referenzgenoms wieder zusammengesetzt [59].

#### Geschichte.

Die Einführung der Pyrosequenzierung im Jahr 1996 durch Mostafa Ronaghi et al. am Royal Institute of Technology in Stockholm war ein wichtiger Schritt auf dem Weg zum NGS [60]. Diese Methode nutzt Echtzeit-Lichtemissionen zur Bestimmung von DNA-Sequenzen. Die erste kommerzielle Nutzung dieses Verfahrens war der „454 Genome Sequencer“, welcher von 454 Life Sciences, einem im Jahr 2000 von Jonathan Rothberg gegründeten Unternehmen, entwickelt und vermarktet wurde. Die Plattform wurde von Roche Diagnostics im Jahr 2007 übernommen [61]. Im Jahr 2006 führte Solexa (später von Illumina übernommen) die Methode „Sequencing by Synthesis“ (SBS) ein, die auch heute noch dominiert. Bei der SBS wird die DNA fragmentiert, mit Adaptern versehen und mit fluoreszenzmarkierten Nukleotiden sequenziert, was eine Hochdurchsatzanalyse von Millionen von Fragmenten gleichzeitig ermöglicht. Im Laufe der Jahre kamen weitere Technologien wie Ion-Torrent und Nanopore-Sequenzierung auf, die sich auf Verbesserungen bei Kosten, Geschwindigkeit und Leselänge konzentrieren [62, 63].

#### Methode.

Obwohl es technisch verschiedene Lösungen gibt, sind die Grundprinzipien des NGS immer ähnlich und bestehen aus den folgenden Schritten [64–66]:

- Probengewinnung und Vorbereitung
- Fragmentation
- Adaptation
- Amplifikation
- Sequenzierung
  - „Illumina sequencing“
  - Ion-Turrent-Verfahren
- Zusammensetzung zu einem kompletten Gen, Exon oder Genom

*Probengewinnung und Vorbereitung*: Nach der Probengewinnung muss die zu untersuchende DNA extrahiert werden. Hier kann es notwendig sein, eine PCR durchzuführen, um die Menge an vorhandener DNA zu vermehren. PCR können ebenfalls hilfreich sein, wenn nur bestimmte Gene oder Exons untersucht werden sollen. Diese Gene oder Exons werden dann vor der eigentlichen NGS-Untersuchung amplifiziert und so selektiert.

*Fragmentation*: Die extrahierte und ggf. amplifizierte DNA wird dann in Bruchstücke aufgeteilt die, je nach Methode etwa 100–500 Basenpaare lang sind. In der ersten kommerziell erhältlichen NGS-Methode, dem 454 Genome Sequencer waren es beispielsweise 100–200 Basenpaare. Aktuelle NGS-Methoden wie die „Oxford Nanopore Technologies“ können routinemäßig Bruchstücke mit einer Länge von 50.000–200.000 Basenpaare sequenzieren. Um die DNA-Bruchstücke herzustellen, gibt es verschiedene Möglichkeiten. Beispielsweise können (Ultra‑)Schall, Nebel oder Flüssigkeiten genutzt werden, um die DNA physisch zu zerkleinern. Alternativ kann die DNA durch Enzyme (DNAasen) oder mittels chemischer Reaktionen zerkleinert werden. Jede dieser Methoden hat Vor- und Nachteile. Welche man verwendet, hängt von der Länge der Basenpaare, der benötigten Genauigkeit und der genutzten NGS-Methode ab.

*Adaptation*: Nachdem man die DNA-Fragmente erstellt hat, werden spezifische Adapter hinzugegeben. Dies sind Oligonukleotide, die mittels passenden DNA-Bruchstücken auf der einen Seite an den Anfang und das Ende von den erstellten Fragmenten binden. Auf der anderen Seite binden diese Adapter an einen Untergrund oder eine Flüssigkeitsperle, abhängig von der gewählten NGS-Methode. Die Adapter haben mehrere Aufgaben: Zum einen sollen sie die Verbindung mit dem NGS-Medium (Flüssigkeitsperle oder sog. Chip) herstellen. Zum anderen enthalten sie einen Primer, der die spätere Amplifikation ermöglicht. Kommen Multiplex-Verfahren zum Einsatz, d. h., dass man mehrere Proben gleichzeitig sequenziert, enthalten die Adapter auch einen Barcode aus speziellen Nukleotidfolgen, mit denen die Bruchstücke hinterher den einzelnen Proben zuzuordnen sind.

*Amplifikation*: Nachdem die Bruchstücke der Ausgangs-DNA mit ihren jeweiligen Adaptern an den Untergrund des jeweiligen NGS-Verfahrens gebunden haben, werden diese Bruchstücke amplifiziert. Es entstehen so an der gleichen Stelle große Mengen an gleichen DNA-Bruchstücken.

*Sequenzierung*: Diese DNA-Bruchstücke werden sequenziert, d. h., es wird analysiert, welche Basen das DNA-Bruchstück bilden. Abhängig vom gewählten NGS-Verfahren gibt es verschiedene Möglichkeiten, wie diese Sequenzierung stattfindet. Beispiele sind: Beim „Illumina sequencing“ sind die Basen an reflektierende Substanzen gebunden. Laser tasten die Proben ab, und erkennen anhand des Reflexionsmusters jede neu eingebaute Base. Beim Ion-Turrent-Verfahren werden die beim Baseneinbau freiwerdenden Protonen und die daraus resultierenden Spannungsunterschiede gemessen. Es gibt allerdings deutlich mehr Methoden, welche alle Vor- und Nachteile haben. Am Ende der Sequenzierung hat man in jedem der Fälle „reads“ also zur fragmentierten DNA passende Basencodes. Die fragmentierte DNA ist damit „erkannt“.

Im letzten Schritt werden diese Bruchstücke wieder zu einem kompletten Gen, Exon oder Genom *zusammengesetzt*. Hier ist eine unglaubliche Rechenleistung notwendig, und es gibt verschiedene Methoden. Wenn man die untersuchte Region kennt, kann der Computer die „reads“ wie bei einem Puzzle entlang des bekannten Genoms aufsetzten und eventuelle Unterschiede feststellen. Ist das Genom unbekannt, muss man sich die Überlappungen zunutze machen. Da die Probe anfangs in zahlreiche Fragmente geteilt wurde, wird es auch immer zahlreiche Überlappungen geben. Wie bei einem Dominospiel werden diese Überlappungen dann gesucht und aneinandergereiht, sodass am Ende die gesamte ursprüngliche DNA entschlüsselt ist.

#### Bedeutung in der Kopf-Hals-Pathologie.

Obwohl das NGS ein sehr potentes diagnostisches Verfahren ist, spielt es aktuell in der klinischen Routine bei der Diagnostik von Kopf-Hals Tumoren noch eine untergeordnete Rolle. Das Verfahren wird jedoch schon in Studien eingesetzt [67]. Blicke in die Nachbardisziplinen, wie beispielsweise die Pneumoonkologie, wo NGS bei nichtresektablen nichtkleinzelligen Lungenkarzinomen (NSCLC) schon routinemäßig durchgeführt wird, um zielgerichtete Checkpointinhibitortherapien einleiten zu können, lassen vermuten, dass dies auch in der Kopf-Hals-Pathologie an Bedeutung gewinnen wird [68, 69].

Das „comprehensive genomic profiling“ (CGP) ermöglicht eine umfassende Analyse der genetischen Veränderungen bei HNO-Tumoren, um gezielte Therapieansätze zu entwickeln. Durch CGP können molekulare Treibermutationen, Genamplifikationen und andere genetische Aberrationen (Deletionen, Tumormutationslast, homologe Rekombinationsdefizienz, Genfusionen/Translokationen) identifiziert werden, die für die Präzisionsmedizin von entscheidender Bedeutung sind. Der Einsatz von CGP bei HNO-Tumoren unterstützt die Auswahl spezifischer zielgerichteter Therapien, wie Tyrosinkinase-Inhibitoren oder Immuntherapeutika. CGP trägt dazu bei, Patienten mit seltenen Mutationen oder Biomarkern zu identifizieren (z. B. *NTRK1‑3*-Genfusionen), die für klinische Studien oder personalisierte Therapieoptionen infrage kommen. Die Integration von CGP in die Routineversorgung könnte die Prognose, die Diagnostik und das Therapieansprechen bei Patienten mit HNO-Tumoren erheblich verbessern [70, 71].

#### Individualisierung vs. Standardisierung.

Das NGS ist ein insoweit voll automatisiertes Verfahren, als dass zur Benutzung nur sehr wenig menschliche Interaktion notwendig ist. Dies gewährleistet einen sehr hohen Grad der Standardisierung. Auch die Auswertung der sehr großen Datenmengen und der Vergleich mit den jeweiligen Referenzgenen geschieht i. d. R. nicht mehr in dem pathologischen Institut vor Ort, sondern wird cloudbasiert von den anbietenden Firmen durchgeführt. Dem gegenüber steht die sehr hohe Individualisierung, da die Ergebnisse auf genomischer Ebene patientenspezifisch sind. Ein aktuelles Problem dieses sehr potenten Verfahrens auf dem Weg zur Routinediagnostik sind die Kosten welche, je nach Art der Untersuchung, 1000 € und mehr schnell übersteigen. Dies zeigt ein wesentliches Problem auf dem Weg der patientenindividuellen Diagnostik, diese ist in aller Regel teuer. Dies trifft umso mehr auf die patientenindividuelle Therapie zu. Hier kann die Medizin allerdings keine alleinige Antwort geben. Wie teuer Individualisierung im Gesundheitssystem sein darf, kann nur von Medizin, Politik und Patienten gemeinsam entschieden werden.

### „Multiscale imaging“ und „drug response profiling“

„Multiscale imaging“ und „drug response profiling“ kombinieren modernste bildgebende Verfahren mit funktionellen Analysen, um die komplexen Wechselwirkungen zwischen Zellprozessen und Medikamentenwirkungen zu verstehen. Durch iterative, indirekte immunfluoreszenzbasierte Verfahren wie 4i-Technologie [72] lassen sich dynamische Veränderungen in Signalwegen auf Einzelzellebene kartieren. Dies ermöglicht es, molekulare Marker zu untersuchen, die mit spezifischen pharmakologischen Interventionen assoziiert sind, und trägt zur Identifikation neuer therapeutischer Ansätze bei.

Ein weiterer Ansatz ist „pharmacoscopy“, ein Verfahren, das die Wirkung von Krebsmedikamenten anhand von Zellabtötung als Endpunkt analysiert [73]. Kombiniert mit genomischen und proteomischen Daten aus Tumorproben, wie in der Tumor Profiler Study beschrieben, können diese Technologien eine ganzheitliche Sicht auf die Tumorbiologie liefern und klinische Entscheidungsprozesse durch präzisere Behandlungsstrategien unterstützen [74].

Zusätzlich bietet die Integration dieser Daten in multidisziplinären Tumorboards die Möglichkeit, standardisierte Diagnostik mit neuen molekularen Erkenntnissen zu verbinden, um personalisierte Therapievorschläge zu entwickeln. So eröffnen diese Ansätze nicht nur ein tieferes Verständnis der Tumorheterogenität, sondern ermöglichen auch die Entdeckung von Biomarkern, die sowohl prädiktiv als auch prognostisch relevant sein können.

Die Kombination dieser Technologien stellt einen bedeutenden Fortschritt im Bereich der personalisierten Medizin dar, indem sie die Brücke zwischen Grundlagenforschung und klinischer Anwendung schlägt und die Therapieergebnisse für Patienten nachhaltig verbessern kann.

## In-vitro-Diagnostik-Richtlinie und Medizinprodukterecht-Durchführungsgesetz

Mit der In-vitro-Diagnostik-Richtlinie (IVDR) der Europäischen Union soll „… ein[en] funktionierender Binnenmarkt für In-vitro-Diagnostika […] sichergestellt werden. Außerdem sind in dieser Verordnung hohe Standards für die Qualität und Sicherheit von In-vitro-Diagnostika festgelegt, durch die allgemeine Sicherheitsbedenken hinsichtlich dieser Produkte ausgeräumt werden sollen.“ Dieser EU-rechtliche Rahmen der Verordnung 2017/746 vom 5. April 2017 regelt in der deutschen Fassung auf 157 Seiten den Umgang mit menschlichen Proben [75]. Mithilfe des Medizinprodukterecht-Durchführungsgesetzes (MPDG) wurde diese EU-Verordnung am 28. April 2020 in nationales Recht umgewandelt [76].

Die Inhalte dieser Richtline und des Gesetzes können im Rahmen dieser Arbeit nicht im Detail wiedergeben werden. Gleichwohl soll der gesetzliche Rahmen, der unbestritten einen wesentlicher Einflussfaktor auf Standardisierungen darstellt, im folgenden Abschnitt aufgezeichnet werden.

### Einteilung in Risikogruppen

Eine wesentliche Neuerung gegenüber den bis dato geltenden Vorschriften ist, dass die Testverfahren in die 4 Klassen A, B, C und D eingeteilt werden, basierend auf dem Risiko, das von den Produkten für Patienten und die öffentliche Gesundheit ausgeht. Hierbei stellt A das niedrigste und D das höchste Risiko da [75].

- **Beispiele für Gruppe A:** 
  - Erzeugnisse für den allgemeinen Laborbedarf,
  - Zubehör ohne kritische Merkmale,
  - Pufferlösungen,
  - Waschlösungen sowie
  - allgemeine Nährmedien und
  - histologische Färbungen [75]
- **Beispiele für Gruppe B:** 
  - Produkte zur Feststellung einer Schwangerschaft,
  - zur Fertilitätsuntersuchung und
  - zur Bestimmung des Cholesterinspiegels und
  - Produkte zum Nachweis von Glukose, Erythrozyten, Leukozyten und Bakterien *im Urin *[75]
- **Beispiele für Gruppe C:** 
  - Einsatz als therapiebegleitende Diagnostika;
  - Einsatz zur Krebsvorsorge, -diagnose oder -Stadieneinteilung;
  - Durchführung von Gentests beim Menschen [75]
- **Beispiele für Gruppe D:** 
  - Nachweis des Vorhandenseins von oder der Exposition gegenüber übertragbaren Erregern in Blut, Blutbestandteilen, Zellen, Geweben oder Organen oder in einem ihrer Derivate, um ihre Eignung für die Transfusion, Transplantation oder Zellgabe zu bewerten,
  - Bestimmung des Infektionsgrads einer lebensbedrohenden Krankheit, dessen Überwachung im Rahmen des Patientenmanagements von entscheidender Bedeutung ist [75].

### Anforderungen für die Risikogruppen

In der pathologischen Diagnostik fallen die Tests also üblicherweise in die Gruppen mit den höchsten Risiken und damit Anforderungen für die Sicherheit, nämlich C und D. Anforderungen, die für alle Risikogruppen gelten, sind [75]:

- **Sicherheits- und Leistungsanforderungen:** Die Produkte müssen sicher sein und die beabsichtigte Leistung zuverlässig erbringen (Anhang I, Abschn. 1)
- **Klinische Evidenz:** Der Hersteller muss ausreichende klinische Daten vorlegen, die die Leistung und Sicherheit des Produkts belegen (Anhang I, Abschn. 1–9).
- **Risikomanagement:** Hersteller müssen ein umfassendes Risikomanagementsystem implementieren (Anhang I, Abschn. 3).

Anforderungen, die zusätzlich für die Risikogruppe C gelten, sind [75]:

- **Klinische Bewertung und Leistungsbewertung (Artikel 56 und Anhang XIII): **Der Hersteller muss eine Leistungsbewertung durchführen, die aus einer analytischen und einer klinischen Bewertung besteht, und die Evidenz muss zeigen, dass das Produkt zuverlässig ist und dessen Ergebnisse reproduzierbar sind.
- **Konformitätsbewertung durch eine benannte Stelle (Artikel 48): **Eine benannte Stelle (z. B. TÜV, DEKRA) muss das Produkt und die technischen Unterlagen bewerten. Der Hersteller muss eine technische Dokumentation vorlegen.
- **Post-Market Surveillance (Artikel 78–81): **Nach dem Inverkehrbringen muss der Hersteller die Leistung und Sicherheit des Produkts kontinuierlich überwachen und dokumentieren. Periodische Sicherheitsberichte (PSUR) müssen an die zuständige Behörde übermittelt werden.

Anforderungen, die zusätzlich für die Gruppe D gelten, sind [75]:

- **Zusätzliche Konformitätsbewertung:** Ein unabhängiges EU-Referenzlabor muss die Leistungsbewertung überprüfen. Für Produkte, die nur „in-house“ verwendet werden, kann auch eine Prüfung durch ein Expertenpanel erforderlich sein.
- **Chargenprüfung:** Jede Charge muss durch eine unabhängige Stelle geprüft werden.
- **Strengere Überwachung nach dem Inverkehrbringen:** Hersteller müssen ein verstärktes Überwachungssystem etablieren, um unerwartete Risiken schnell zu erkennen.

#### Bedeutung für die Pathologie.

Auch wenn sich der Großteil der Anforderungen dieser Verordnung an die Hersteller richtet, so dürfte dieser Mehraufwand mittelbar über Preissteigerungen an die Verbraucher weitergegeben werden. Außerdem bedeutet er für die Verbraucher einen bedeutenden Mehraufwand bei Dokumentation, Meldepflichten und Integration in vorhandene Qualitätsmanagement(QM)-Systeme [77]. Wesentliche Anforderungen betreffen auch selbst entwickelte Testverfahren, die sog. „lab-developed tests“ (LDT), welche v. a. in der Tumordiagnostik zum Einsatz kommen. Hier gelten erhöhte Dokumentations- und Validierungspflichten [76, 77]. Insgesamt stellt die Umsetzung der Verordnung neben den Kernaufgaben in der Krankenversorgung, der Lehre und Forschung eine zusätzliche große personelle und zeitliche Herausforderung dar [78].

## Digitalisierung der Pathologie

### Einsatz in der Gewebediagnostik

**Abb. 3:**
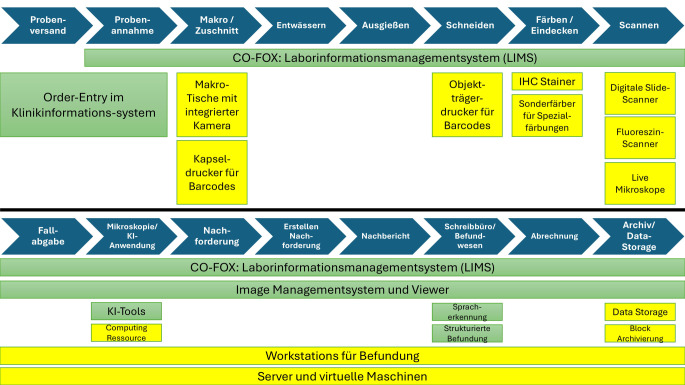
Digitaler Workflow der Gewebediagnostik an der Universitätsmedizin Frankfurt. *Blau *Weg der Proben; *grün *benötigte Software; *gelb *benötigte Hardware, *CO-FOX* Fa. Co-Fox GmbH, Celle, Deutschland; *IHC* Immunhistochemie; *KI* künstliche Intelligenz

In den letzten Jahren wird der Fokus bei den Arbeitsabläufen und der Befundung auf die fortschreitende Digitalisierung gesetzt. Jeder Aspekt der Prozesskette (Abb. 3) kann hier durch Digitalisierung potenziell transparenter und/oder effizienter gestaltet werden. Beispielsweise kann schon bei der Probenentnahme im OP die Anforderung digital im Klinikinformationssystem und nicht mehr herkömmlich auf Durchschlagpapier gestellt werden. Beim Versenden der Proben zum pathologischen Institut kann ein Probentracking analog zu den Paketdiensten verwendet werden, damit kann eine Nachverfolgung und Prävention von Probenverlusten erreicht werden.

Der Prozess der digitalen Pathologie beginnt mit dem Probeneingang und umfasst verschiedene Schritte von der Probenvorbereitung bis zur Archivierung. Zu den Schritten gehören [79, 80]:

1. Probenversand und -annahme: Erfassung und Zuordnung der Probe mittels Order-Entry-Systemen.
2. Makroskopie- bzw. Zuschnitt-Arbeiten: Nutzung integrierter Kameras und Barcode-Drucker für Probenkennzeichnung und Dokumentation.
3. Histologische Aufbereitung: Entwässern, Ausgießen, Schneiden, Färben und Eindecken von Gewebeschnitten unter standardisierten Bedingungen.
4. Digitalisierung: Scanner und Live-Mikroskope ermöglichen hochauflösende digitale Bildgebung für KI-gestützte Analysen.
5. Befundung: KI-Tools und strukturierte Befundsysteme unterstützen Pathologen bei der Analyse und Dokumentation von Ergebnissen.
6. Archivierung und Speicherung der Daten: Digitale Daten werden in umfangreichen Speichersystemen organisiert und gesichert.

Ergänzend zur Prozesskette werden unterstützende Systeme wie das Co-Fox-LIMS (Laborinformationssystem) und die Software AetherAI (Fa. AetherAI, Taipei City, Taiwan) für Bildmanagement (IMS) eingesetzt, um eine nahtlose Integration und Analyse zu gewährleisten. IT-Sicherheit und Serverinfrastrukturen gewährleisten die Zuverlässigkeit der Arbeitsumgebung.

Im eigentlichen Sinne bezeichnet die digitale Pathologie aber den Prozess der Bildbetrachtung und Archivierung. Der aktuelle Standard der meisten pathologischen Institute ist ein Betrachten von herkömmlich oder immunhistochemisch gefärbten Objektträgern im Lichtmikroskop.

Hier kann eine Analogie zur Radiologie gezogen werden, wo es bis etwa 2010 auch sehr weit verbreitet war, Röntgenbilder physisch an Bildbetrachtern zu befunden. Auch hier erfolgte die fast flächendeckende Umstellung auf digitale Bildbetrachtung.

Der Hauptunterschied zur Pathologie ist jedoch, dass zumindest in den Schnittbildgebungen (Computertomographie [CT] und Magnetresonanztomographie [MRT]) die Bilder schon immer digital erstellt waren und früher ausgedruckt wurden. Es fiel hier also ein Arbeitsschritt weg.

Histologische und immunhistochemische Untersuchungen werden auch mittelfristig mittels Schnitten und auf Objektträgern stattfinden, sodass hier ein Scannen der Objektträger in maximal möglicher Bildqualität notwendig ist.

Neben der hochwertigen Digitalisierung dieser Objektträger sind die folgenden Punkte für die Umsetzung einer digitalen Pathologie wichtig [79, 80]:

- digitales Prozessmanagement,
- digitale Diagnostik,
- digitale Kommunikation,
- digitale Fort- und Weiterbildung,
- Datensicherheit.

Mit der Umsetzung soll eine Reihe von Vorteilen erreicht werden:

- **Optimierung von Arbeitsabläufen:** Digitale Systeme verbessern die Effizienz und erlauben eine automatisierte Bearbeitung repetitiver Aufgaben, wie Längenmessungen oder die Quantifizierung immunhistochemischer Färbungen [81].
- **Steigerung der Fallzahlen:** Ein effizienteres Arbeiten und eine Steigerung der Fallzahl ist auch gerade vor dem Hintergrund des Fachkräftemangels, der auch in der Pathologie herrscht, wichtig [81, 82].
- **Effizientere Archivierung:** Älteres Patientenmaterial kann schneller und einfacher wiedergefunden werden [83].
- **Schnellere Kommunikation:** Digitale Objektträger können schneller zwischen Standorten geteilt werden, was die Einholung von Zweitmeinungen erleichtert [83].
- **Ortsunabhängigkeit:** Pathologen sind nicht mehr an das Labor gebunden und können „remote“ arbeiten [84].

Iwuajoku et al. haben den Prozess der Umstellung am pathologischen Institut der Universitätsklinik München wissenschaftlich begleitet. Dabei identifizierten sie die folgenden Punkte, welche zu Schwierigkeiten führten [81]:

- technische Probleme mit den Scannern,
- Qualitätsprobleme der Objektträger (zu viel Material, falsche Barcodes usw.),
- Fehler im Betriebsablauf durch neue Prozesse und unzureichende Kommunikation.

Ein der Digitalpathologie zwangsläufig nachgeschalteter Schritt ist die „computational pathology“, welche digitale Gewebeschnitte mithilfe von Algorithmen analysiert. So sollen Prognosen oder Therapieansprechen besser vorhergesagt werden und quantitative Daten generiert werden [4]. Die Standardisierung digitaler Arbeitsabläufe für Effizienz und Datenmanagement bietet so unmittelbar ein Potenzial für eine stärkere Individualisierung durch fortschrittliche Bildanalyse und datengesteuerte Erkenntnisse. Insgesamt ist davon auszugehen, dass das Potenzial der digitalen Pathologie dazu geeignet ist, die Arbeitsweisen in der Pathologie grundlegend zu reformieren und den Austausch zwischen Klinikern und Pathologen zu verbessern.

### Künstliche Intelligenz in der Diagnostik

Die digitale und „computational pathology“ hat in den letzten Jahren erheblich zur Transformation der pathologischen Diagnostik beigetragen, sie bewirken eine transformative Ära. Die digitale Pathologie basiert auf der Digitalisierung histologischer Schnitte zu hochauflösenden Ganzschnittbildern („whole-slide images“, WSI), die Grundlage für eine datengetriebene Analyse sind. „Computational pathology“ erweitert diesen Ansatz durch Algorithmen des maschinellen Lernens und der künstlichen Intelligenz (KI), um diagnostische Prozesse zu automatisieren und zu erweitern.

Ein Meilenstein in diesem Bereich war die Entwicklung computerunterstützter („computational“) Ansätze für die Pathologie, wie sie erstmals auf der MICCAI-Konferenz etabliert wurden [85]. Hierbei definierten Forscher wie Wild und Fuchs zentrale Frameworks, die moderne Deep-Learning-Methoden auf große histopathologische Bilddaten anwenden und durch „multiple-instance learning“ eine interpretierbare Klassifikation ermöglichen. Diese Ansätze bieten klinisch relevante Einblicke, wie etwa in der molekularen Subtypisierung von Tumoren und der Überlebensvorhersage bei Krebspatienten. Besonders hervorzuheben ist die Fähigkeit dieser Modelle, mikroskopische Muster zu identifizieren, die menschliche Beobachter nicht wahrnehmen können, was eine präzisere Diagnostik ermöglicht.

Ein weiterer Fortschritt ist die Anwendung von Foundation-Modellen, großen vortrainierten neuronalen Netzwerken, die domänenübergreifend arbeiten können. Diese Modelle, wie sie auch in der Radiologie und Molekularbiologie angewendet werden, zeigen großes Potenzial für die Pathologie, indem sie komplexe Bild- und Metadaten integrieren. Studien wie die von Woerl et al. und Jurmeister et al. demonstrieren, dass diese Modelle die molekulare Charakterisierung von Tumoren wie Blasen- oder Lungenkrebs signifikant verbessern können [86, 87].

Darüber hinaus sind Open-Source-Plattformen wie QuPath entscheidend, da sie eine breite Anwendung und Weiterentwicklung computerunterstützter Methoden ermöglichen [88]. Solche Tools fördern den Einsatz von KI in der Forschung und diagnostischen Routinen, wie es auch in der AG Wild implementiert wird.

Zusammenfassend steht die digitale Pathologie und „computational pathology“ an der Schwelle zu einer datengetriebenen Medizin, die diagnostische Genauigkeit und Effizienz steigern kann. Durch die Integration fortschrittlicher KI-Modelle und die fortlaufende Standardisierung digitaler Prozesse wird die Pathologie zunehmend zu einer interdisziplinären Wissenschaft, die personalisierte Medizin aktiv vorantreibt.

### Anwendung bei HNO-Tumoren

Die computergestützte Pathologie revolutioniert die Diagnostik und Behandlung von Kopf-Hals-Tumoren, insbesondere durch den Einsatz von maschinellem Lernen und molekularbiologischen Ansätzen wie DNA-Methylierungsprofilen. Diese Technologien ermöglichen präzisere Diagnosen, die über herkömmliche histologische Analysen hinausgehen.

Ein wegweisendes Beispiel ist die Arbeit von Jurmeister et al., bei der ein Deep-Learning-Ansatz auf DNA-Methylierungsdaten angewendet wurde, um zwischen primären Lungentumoren und Kopf-Hals-Metastasen zu unterscheiden [87]. Die Methode erreicht eine Genauigkeit von über 99 % und ermöglicht eine differenzierte Klassifikation, was essenziell für die Wahl der Therapie ist, insbesondere bei der Abgrenzung von Metastasen gegenüber Zweittumoren in der Lunge. Diese Ergebnisse unterstreichen die Bedeutung von KI-gestützter Diagnostik, um die Überlebenschancen der Patienten durch gezielte Behandlungsansätze zu verbessern [87].

Ein weiteres Beispiel betrifft die Klassifikation von seltenen sinunasalen undifferenzierten Karzinomen (SNUC). Hier konnte durch DNA-Methylierungsanalysen und maschinelles Lernen eine Unterteilung in 4 molekulare Gruppen erreicht werden, die prognostisch und therapeutisch relevant sind. Einige Gruppen zeigen überraschend gute Verläufe trotz eines aggressiven Erscheinungsbilds, während andere eine ungünstigere Prognose aufweisen. Diese Erkenntnisse eröffnen neue Möglichkeiten für personalisierte Therapien und die Entwicklung spezifischer molekularer Zielstrukturen [89].

Zusammenfassend zeigt die Anwendung von „computational pathology“ bei HNO-Tumoren das enorme Potenzial, durch Integration von KI und molekularer Pathologie präzisere und individuell abgestimmte Behandlungen zu ermöglichen. Die Forschung in diesem Bereich markiert einen bedeutenden Schritt in Richtung personalisierter Onkologie.

## Präzisionsmedizin und genomische Medizin

### Biomarker

Präzisionsmedizin oder die häufig synonym gebrauchten Termini individualisierte, personalisierte, geschichtete Medizin („stratified medicine“) bezeichnet den Versuch, Patienten unter Verwendung diagnostischer Tests oder Verfahren in Gruppen bezüglich des Krankheitsrisikos oder des erwarteten Behandlungserfolgs einzuteilen [90]. Ziel dieses Ansatzes ist es, dass Patienten von gezielteren und wirksameren Behandlungen profitieren [90].

Zur Gruppierung werden i. d. R. Biomarker eingesetzte, wobei die US-amerikanische Food and Drug Administration (FDA) Biomarker als „ein definiertes Merkmal, das als Indikator für normale biologische Prozesse, pathogene Prozesse oder Reaktionen auf eine Exposition oder Intervention gemessen wird“ definiert [91]. Man unterscheidet grundsätzlich diagnostische von prognostischen und prädiktiven Biomarkern, wobei prognostische Biomarker den Einfluss einer Therapie voraussagen sollen und prädiktive Biomarker den Krankheitsverlauf unabhängig von der Therapie abschätzen [91, 92].

Das generelle Konzept, Patienten anhand von sicht- oder messbaren Markern zu gruppieren, ist vermutlich so alt wie die Medizin selbst. Hippokrates werden die Worte „Es ist wichtiger zu wissen, welche Person eine Krankheit hat, als zu wissen, welche Krankheit eine Person hat“ zugeschrieben. Auch in der modernen Medizin teilen wir Erkrankungen ständig in Gruppen ein, um die Behandlung zu verbessern. Beispielsweise überlegen wir uns bei jeder Otitis, ob diese bakteriell oder viral ist, um eine „gezielte“ Antibiotikabehandlung einzuleiten.

### Genomsequenzierungen

Neu ist der Umfang, in dem es möglich ist, patientenspezifische Unterschiede auf molekularer und genetischer Ebene nachzuvollziehen. Hier spielt v. a. das „next-generation sequencing“ eine wichtige Rolle. Das führt dazu, dass der Begriff personalisierte Medizin heute auch häufig nur für den Teilbereich der Genomanalyse und nicht für das gesamte Behandlungskonzept genutzt wird. Ursprünglich stellt die molekulare Analyse der Erkrankung aber nur einen Teilbereich des Behandlungskonzepts der personalisierten Medizin dar. In der Kopf-Hals-Onkologie spielen heute schon die beschriebene Unterscheidung des HPV- und PD-L1-Status eine wichtige klinische Rolle. Weitere Biomarker wie beispielsweise die Tumor-DNA oder HPV-DNA in „liquid biopsies“ werden vermutlich bald folgen [39]. Zudem wird der breite Ansatz von Genomsequenzierungen zur umfassenden Mutationsanalyse und Erstellung individueller molekularer Profile vermutlich dafür sorgen, dass Medikamente wie beispielsweise Immuncheckpointinhibitoren noch zielgerichteter entsprechend bestimmter genetischer Merkmale eingesetzt werden.

### Individualisierter chirurgischer Ansatz

Weitere Aspekte sind ein individualisierter chirurgischer Ansatz mit detaillierter Operationsplanung, neuen Methoden der Randschnittbeurteilung wie beispielsweise die konfokale Fluoreszenzmikroskopie, welche histologische Proben mittels Laserscan patientennah digitalisieren kann und so zum einen eine Remoteauswertung möglich macht und zum anderen für die Chirurgen eine Art „Point-of-Care-Pathologie“ ermöglicht [93, 94].

Dies alles muss allerdings auch immer unter dem Kosten-Nutzen-Aspekt gesehen werden: Aktuell wird in Deutschland von den Kostenträgern bei Weitem nicht alles, was möglich ist, auch bezahlt. Gleichzeitig muss – bezogen auf den wirtschaftlichen Ressourceneinsatz – auch nicht alles, was möglich ist, unbedingt durchgeführt werden. Hier stehen adäquate gesundheitsökonomische Untersuchungen und Bewertungen aus. Medizin und Politik sind gefordert, in den Dialog zu treten, um diese unbequeme Diskussion zu führen. Der Ansatz der Präzisionsmedizin kann jedoch helfen, Kosten zu reduzieren, wenn durch gute Screening-Untersuchungen auf sonst notwendige invasivere Diagnostik- und Behandlungsmethoden verzichtet werden kann.

Auch hier gilt, dass nur durch die enge Zusammenarbeit zwischen Pathologen, Klinikern, Bioinformatikern, Gesundheitsökonomen und weiteren Experten die optimale Nutzung von Daten und Biomarkern zum Wohle der Patienten gelingen kann.

## Ausblick

Die chirurgische Pathologie in der Hals-Nasen-Ohren-Medizin (HNO) befindet sich in einer Phase des Umbruchs, geprägt von regulatorischen Veränderungen, technischen Innovationen und der wachsenden Bedeutung präzisionsmedizinischer Ansätze. Die zentralen Aspekte dieser Entwicklung – IVDR, digitale Pathologie und Präzisionsmedizin – werfen gemeinsam mit den technischen Besonderheiten der pathologischen Untersuchungen spezifische Herausforderungen auf, bieten aber auch erhebliche Vorteile. Die technischen Besonderheiten der in der Pathologie gebräuchlichen Tests machen eine Standardisierung über die Grenzen des eigenen Labors hinweg schwierig. Eine Reihe von Methoden wurde entwickelt, um diese Schwierigkeiten zu überwinden.

### Qualitätssicherungsmaßnahmen

Zum einen werden immer speziellere Qualitätssicherungsmaßnahmen, welche einen hohen Aufwand an Material und Personal erfordern, implementiert, wie beispielsweise Ringversuche, bei denen eine identische Probe von einer zentralen Stelle an mehrere teilnehmende Labore verschickt und anschließend unabhängig analysiert wird. Die Ergebnisse werden dann mit Sollwert und/oder den Ergebnissen anderer Labore verglichen. Das Ziel ist, die Leistungsfähigkeit der Tests zu überprüfen, methodische Unterschiede aufzudecken und die Genauigkeit der Analysen zu verbessern. Außerdem werden spezifische Scores genutzt, welche auf Verhältnisse von Untersuchungsergebnissen unabhängig vom Ort eingehen.

### Sicherheits- und Leistungsnachweise

Der gesetzliche Rahmen, in dem das zu geschehen hat, wird von der IVDR geregelt. Hier kommen zusätzliche regulatorische Anforderungen insbesondere in der Tumordiagnostik hinzu. Die Einstufung diagnostischer Tests in Risikoklassen C und D erfordert Sicherheits- und Leistungsnachweise, darunter klinische Validierungen, Risikomanagement und regelmäßige Überwachung nach Markteinführung. Besonders herausfordernd ist die Einhaltung der Vorgaben für „lab-developed tests“ (LDT), die in der Tumorpathologie häufig Anwendung finden.

### Zweitmeinungen

Die Einführung von digitaler Pathologie wird die Arbeitsweise von Pathologen vermutlich nachhaltig verändern. Nach dem Scannen der Objektträger können diese digital archiviert und ortsunabhängig analysiert werden. Dies ermöglicht nicht nur schnelleren Zugriff auf Patientenmaterial, sondern erleichtert auch den Austausch zwischen Kliniken und das Einholen von Zweitmeinungen. Zukünftig könnten Pathologen histologische Bilder ähnlich wie Radiologen in Tumorkonferenzen präsentieren und interdisziplinär besprechen, was eine stärkere Verknüpfung von Diagnostik und Therapieplanung ermöglicht. Zudem ist die Digitalisierung von Bilddaten Voraussetzung für automatisierte Prozesse wie KI-gestützte Bildanalyse. Entlastung schafft die Digitalisierung auch bei repetitiven Aufgaben wie der Quantifizierung von immunhistochemischen Färbungen. Gleichzeitig stellen technische Herausforderungen wie die Standardisierung von Scannern, die Qualität der Objektträger und die Integration neuer Arbeitsabläufe in bestehende Systeme eine Hürde dar. Insgesamt ist die digitale Pathologie durch Standardisierung ein Schritt in Richtung Individualisierung, indem sie durch fortschrittliche Bildanalysen patientenspezifische Erkenntnisse liefert und den interdisziplinären Austausch, der v. a. in der Präzisionsmedizin notwendig ist, fördert.

### Molekulare Profile von Tumoren

Zudem sorgt die computergestützte Pathologie in der Diagnostik und Behandlung von Tumoren, insbesondere durch den Einsatz von maschinellem Lernen und molekularbiologischen Ansätzen wie DNA-Methylierungsprofilen, für einen revolutionär neuen Ansatz.

Beispiele für schon jetzt genutzte Präzisionsmedizin in der Kopf-Hals-Onkologie sind Biomarker wie HPV-Status und PD-L1-Expression. Der Nachweis von p16-Expression durch Immunhistochemie hat sich zur Identifikation von HPV-assoziierten Tumoren etabliert und wird routinemäßig eingesetzt. Die PD-L1-Diagnostik beeinflusst direkt die Entscheidung über den Einsatz von Checkpointinhibitoren wie Nivolumab oder Pembrolizumab in Rezidivsituationen.

Mit der zunehmenden Verfügbarkeit von „next-generation sequencing“ (NGS) wird es möglich, umfassende molekulare Profile von Tumoren zu erstellen. In der Pneumologie ist dies bei NSCLC bereits Routine. Eine ähnliche Entwicklung, spätestens wenn geeignete Therapieziele für Immuncheckpointinhibitoren identifiziert sind, darf auch für die HNO-Pathologie erwartet werden. „Liquid biopsies“, die zirkulierende Tumor-DNA im Blut analysieren, könnten invasive Diagnostikmethoden ergänzen und die Überwachung des Therapieerfolgs erleichtern. Gleichzeitig bleibt der Kosten-Nutzen-Aspekt eine Herausforderung, da viele innovative Verfahren derzeit nicht von den Kostenträgern übernommen werden.

Am Beispiel der Pathologie lässt sich zeigen, dass die Standardisierung von Verfahren letztlich einer Verbesserung der individuellen Behandlung dient. Zugleich lassen sich aber auch die Herausforderungen ablesen: Trotz umfassender Regularien sowie einer Laborumgebung mit digitaler Unterstützung ist die Standardisierung sehr zeit- und kostenaufwendig. Eine enge interdisziplinäre Zusammenarbeit zwischen Pathologen, IT-Experten und Klinikern wird entscheidend sein, um die Potenziale dieser Entwicklungen voll auszuschöpfen. Will man ähnliche Standardisierungsansätze beispielsweise in einem operativen Umfeld wie der HNO-Chirurgie umsetzen, darf der Aufwand als mindestens gleichwertig, wenn nicht höher eingeschätzt werden, da hier die „menschliche Komponente“ eine viel größere Rolle spielt als beispielsweise bei Laboruntersuchungen.

## Fazit für die Praxis

- Die Standardisierung von Verfahren dient einer Verbesserung der individuellen Behandlung, ist aber in der Etablierung häufig zeit- und kostenaufwendig.
- Mit der zunehmenden Verfügbarkeit von „next-generation sequencing“ (NGS) wird es möglich, umfassende molekulare Profile von Tumoren zu erstellen.
- Beispiele für schon jetzt genutzte Präzisionsmedizin in der Kopf-Hals-Onkologie sind Biomarker wie der Status hinsichtlich humaner Papillomaviren (HPV) und die PD-L1-Expression.
- Die computergestützte Pathologie revolutioniert die Diagnostik und Behandlung von Tumorerkrankungen, insbesondere durch den Einsatz von maschinellem Lernen und molekularbiologischen Ansätzen wie DNA-Methylierungsprofilen.
- „Multiscale imaging“ und „drug response profiling“ kombinieren modernste bildgebende Verfahren mit funktionellen Analysen, um die komplexen Wechselwirkungen zwischen Zellprozessen und Medikamentenwirkungen zu verstehen.
